# Supplementary material for: An open-source framework for large-scale, flexible evaluation of biomedical text mining systems
Source: J Biomed Discov Collab. 2008 Jan 29;3:1. doi: 10.1186/1747-5333-3-1 (PMC2276192; doi:10.1186/1747-5333-3-1)
Supplement: Additional file 1 — Evaluation platform source code. The additional file contains the software evaluation framework discussed in this paper. The most current release can be downloaded from SourceForge.net [1]. See the accompanying README file for instructions on installation and use. Also included in the distribution is a collection of UIMA wrappers for some commonly used BioNLP tools and annotated corpora. [file 1747-5333-3-1-S1.zip › bionlp_uima_repository-1.0.1/docs/api/edu/uchsc/ccp/uima/cc/summary/comparison/AnnotationComparator_CC.html]

AnnotationComparator\_CC (Center for Computational Pharmacology Util/UIMA API)


|  |  |  |
| --- | --- | --- |
| **Overview** \| **Package** \| **Class** \| **Tree** \| **Deprecated** \| **Index** \| **Help** | |  |
| PREV CLASS   **NEXT CLASS** | **FRAMES**    **NO FRAMES**     **All Classes** |  |
| SUMMARY: NESTED \| FIELD \| CONSTR \| METHOD | DETAIL: FIELD \| CONSTR \| METHOD |  |


---


## edu.uchsc.ccp.uima.cc.summary.comparison Class AnnotationComparator\_CC

```
java.lang.Object
  org.apache.uima.resource.Resource_ImplBase
      org.apache.uima.resource.ConfigurableResource_ImplBase
          org.apache.uima.collection.CasConsumer_ImplBase
              edu.uchsc.ccp.uima.cc.summary.comparison.AnnotationComparator_CC
```

**All Implemented Interfaces:**: org.apache.uima.collection.base\_cpm.CasObjectProcessor, org.apache.uima.collection.base\_cpm.CasProcessor, org.apache.uima.collection.CasConsumer, org.apache.uima.resource.ConfigurableResource, org.apache.uima.resource.Resource

---

``` public class AnnotationComparator_CC extends org.apache.uima.collection.CasConsumer_ImplBase implements org.apache.uima.collection.base_cpm.CasObjectProcessor ```

**Author:**
:   williamb

---

| **Field Summary** | |
| --- | --- |
| `static String` | `PARAM_ANNOTATION_OUTPUT_FILE`             if filled, TP, FP, and FN annotations will be printed to file |
| `static String` | `PARAM_CONFIG_FILE` |
| `static String` | `PARAM_USE_IDENTICAL_MENTION_COMPARATOR` |
| `static String` | `PARAM_USE_IGNORE_SPAN_COMPARATOR` |
| `static String` | `PARAM_USE_OVERLAP_SPAN_COMPARATOR` |
| `static String` | `PARAM_USE_SHARED_END_SPAN_COMPARATOR` |
| `static String` | `PARAM_USE_SHARED_START_OR_END_SPAN_COMPARATOR` |
| `static String` | `PARAM_USE_SHARED_START_SPAN_COMPARATOR` |
| `static String` | `PARAM_USE_STRICT_SPAN_COMPARATOR` |

**Fields inherited from interface org.apache.uima.resource.Resource**

`PARAM_AGGREGATE_SOFA_MAPPINGS, PARAM_CONFIG_PARAM_SETTINGS, PARAM_PERFORMANCE_TUNING_SETTINGS, PARAM_RESOURCE_MANAGER, PARAM_UIMA_CONTEXT`


**Constructor Summary**

`AnnotationComparator_CC()`


| **Method Summary** | |
| --- | --- |
| `void` | `collectionProcessComplete(org.apache.uima.util.ProcessTrace aTrace)`             Print the comparison results |
| `void` | `initialize()`             Initializes this CAS Consumer with the parameters specified in the descriptor. |
| `void` | `processCas(org.apache.uima.cas.CAS aCAS)` |

**Methods inherited from class org.apache.uima.collection.CasConsumer\_ImplBase**

`batchProcessComplete, destroy, getProcessingResourceMetaData, initialize, isReadOnly, isStateless, processCas, reconfigure, typeSystemInit`

**Methods inherited from class org.apache.uima.resource.ConfigurableResource\_ImplBase**

`getConfigParameterValue, getConfigParameterValue, setConfigParameterValue, setConfigParameterValue`

**Methods inherited from class org.apache.uima.resource.Resource\_ImplBase**

`getCasManager, getLogger, getMetaData, getResourceManager, getUimaContext, getUimaContextAdmin, setLogger, setMetaData`

**Methods inherited from class java.lang.Object**

`clone, equals, finalize, getClass, hashCode, notify, notifyAll, toString, wait, wait, wait`

**Methods inherited from interface org.apache.uima.collection.base\_cpm.CasObjectProcessor**

`processCas, typeSystemInit`

**Methods inherited from interface org.apache.uima.collection.base\_cpm.CasProcessor**

`batchProcessComplete, getProcessingResourceMetaData, isReadOnly, isStateless`

**Methods inherited from interface org.apache.uima.resource.ConfigurableResource**

`getConfigParameterValue, getConfigParameterValue, setConfigParameterValue, setConfigParameterValue`

**Methods inherited from interface org.apache.uima.resource.Resource**

`getLogger, getMetaData, getResourceManager, getUimaContext, getUimaContextAdmin, setLogger`

**Field Detail**

### PARAM\_CONFIG\_FILE

```
public static final String PARAM_CONFIG_FILE
```

**See Also:**: Constant Field Values

---


### PARAM\_USE\_STRICT\_SPAN\_COMPARATOR

```
public static final String PARAM_USE_STRICT_SPAN_COMPARATOR
```

**See Also:**: Constant Field Values

---


### PARAM\_USE\_OVERLAP\_SPAN\_COMPARATOR

```
public static final String PARAM_USE_OVERLAP_SPAN_COMPARATOR
```

**See Also:**: Constant Field Values

---


### PARAM\_USE\_SHARED\_START\_SPAN\_COMPARATOR

```
public static final String PARAM_USE_SHARED_START_SPAN_COMPARATOR
```

**See Also:**: Constant Field Values

---


### PARAM\_USE\_SHARED\_END\_SPAN\_COMPARATOR

```
public static final String PARAM_USE_SHARED_END_SPAN_COMPARATOR
```

**See Also:**: Constant Field Values

---


### PARAM\_USE\_SHARED\_START\_OR\_END\_SPAN\_COMPARATOR

```
public static final String PARAM_USE_SHARED_START_OR_END_SPAN_COMPARATOR
```

**See Also:**: Constant Field Values

---


### PARAM\_USE\_IGNORE\_SPAN\_COMPARATOR

```
public static final String PARAM_USE_IGNORE_SPAN_COMPARATOR
```

**See Also:**: Constant Field Values

---


### PARAM\_USE\_IDENTICAL\_MENTION\_COMPARATOR

```
public static final String PARAM_USE_IDENTICAL_MENTION_COMPARATOR
```

**See Also:**: Constant Field Values

---


### PARAM\_ANNOTATION\_OUTPUT\_FILE

```
public static final String PARAM_ANNOTATION_OUTPUT_FILE
```

:   if filled, TP, FP, and FN annotations will be printed to file

    **See Also:**: Constant Field Values


**Constructor Detail**

### AnnotationComparator\_CC

```
public AnnotationComparator_CC()
```


**Method Detail**

### initialize

```
public void initialize()
                throws org.apache.uima.resource.ResourceInitializationException
```

:   Initializes this CAS Consumer with the parameters specified in the descriptor.

    :   **Overrides:**: `initialize` in class `org.apache.uima.collection.CasConsumer_ImplBase`
    :   **Throws:**: `org.apache.uima.resource.ResourceInitializationException` - if there is error in initializing the resources

---


### processCas

```
public void processCas(org.apache.uima.cas.CAS aCAS)
                throws org.apache.uima.resource.ResourceProcessException
```

:   **Specified by:**: `processCas` in interface `org.apache.uima.collection.base_cpm.CasObjectProcessor`
:   **Throws:**: `org.apache.uima.resource.ResourceProcessException`

---


### collectionProcessComplete

```
public void collectionProcessComplete(org.apache.uima.util.ProcessTrace aTrace)
                               throws org.apache.uima.resource.ResourceProcessException,
                                      IOException
```

:   Print the comparison results

    :   **Specified by:**: `collectionProcessComplete` in interface `org.apache.uima.collection.base_cpm.CasProcessor` **Overrides:**: `collectionProcessComplete` in class `org.apache.uima.collection.CasConsumer_ImplBase`
    :   **Throws:**: `org.apache.uima.resource.ResourceProcessException`: `IOException`


---


|  |  |  |
| --- | --- | --- |
| **Overview** \| **Package** \| **Class** \| **Tree** \| **Deprecated** \| **Index** \| **Help** | |  |
| PREV CLASS   **NEXT CLASS** | **FRAMES**    **NO FRAMES**     **All Classes** |  |
| SUMMARY: NESTED \| FIELD \| CONSTR \| METHOD | DETAIL: FIELD \| CONSTR \| METHOD |  |


---
